# Desiccation Stress Tolerance in *Porphyra* and *Pyropia* Species: A Latitudinal Analysis along the Chilean Coast

**DOI:** 10.3390/plants12010012

**Published:** 2022-12-20

**Authors:** Loretto Contreras-Porcia, Andrés Meynard, Florentina Piña, Manoj Kumar, Carlos Lovazzano, Alejandra Núñez, María Rosa Flores-Molina

**Affiliations:** 1 Departamento de Ecología y Biodiversidad, Facultad de Ciencias de la Vida, Universidad Andres Bello, República 440, Santiago 8370251, Chile; 2 Centro de Investigación Marina Quintay (CIMARQ), Facultad de Ciencias de la Vida, Universidad Andres Bello, Quintay 2531015, Chile; 3 Center of Applied Ecology and Sustainability (CAPES), Santiago 8331150, Chile; 4 Instituto Milenio en Socio-Ecología Costera (SECOS), Santiago 8370251, Chile; 5 Climate Change Cluster, Faculty of Science, University of Technology Sydney, Sydney, NSW 2007, Australia; 6 Instituto de Bioquímica y Microbiología, Facultad de Ciencias, Universidad Austral de Chile, Valdivia 5110566, Chile; 7 Millennium Institute for Integrative Biology (iBio), Santiago 8331150, Chile

**Keywords:** desiccation stress, latitudinal distribution, Laver, Luche, Nori, ocean warming, oxidative stress, red seaweeds, antioxidants, reactive oxygen species

## Abstract

One of the most important factors regulating the distribution and abundance of seaweeds is desiccation, triggered mainly by tidal changes and climatic variation. *Porphyra* and *Pyropia* species have evolved multiple strategies to tolerate desiccation stress; however, how these tolerance strategies differ in these species inhabiting different latitudes is still unknown. In this context, we analyzed, in situ, the physiological responses of these species (collected from 18° S to 41° S along the Chilean coast) to desiccation stress using biochemical and molecular analyses. The hyper-arid terrestrial climate of northern Chile, with high evaporation and lack of constant rain determines a very steep increase in desiccation stress in the upper intertidal during low tide for these species. Accordingly, the results showed that, in comparison with the southernmost populations, the *Porphyra*/*Pyropia* species from the north zone of Chile (18°–30° S) exhibited higher contents of lipoperoxide and carbonyls (1.6–1.9 fold) together with higher enzymatic activities, including ascorbate peroxidase, catalase, peroxiredoxin, and thioredoxin (2–3-fold). In addition, a substantial expression of *cat*, *prx*, and *trx* transcripts during desiccation was demonstrated, mainly in the northernmost populations. These results provide evidence of (i) significant activation of antioxidant enzymes and transcripts (principally *cat* and *prx*); (ii) participation of phenolic antioxidant compounds as a highly plastic physiological strategy to cope with desiccation; and (iii) the activation of the tolerance responses was affected by species latitudinal distribution. Thus, for the first time, this study integrated the biochemical and genetic responses of diverse *Porphyra*/*Pyropia* species to better understand their physiological dynamics of tolerance over a wide latitudinal range.

## 1. Introduction

Seaweeds are at the base of the marine food chain and show different distribution patterns along the rocky intertidal zone, between the upper and lower limits. At the upper limit, the distribution pattern is determined mainly by a variety of stressful abiotic conditions, among which is desiccation stress (e.g., [1,2]), induced by tidal changes and local climatic conditions. Other fluctuating and stressful environmental factors that could determine the presence of seaweeds, especially in the high intertidal, are salinity, radiation, temperature, pollutants, and likely, as occurs with salt marshes and mangroves, sediment anoxia [1,3]. Even though acclimation to these environmental stressors involves similar physiological changes, such as antioxidant enzymes and non-enzymatic antioxidants, organisms have also evolved specific constitutive or induced responses to each of these stressors. For example, to improve their salinity tolerance, some green algae have evolved several adaptations, such as genes and transporters promoting cytosolic K^+^/Na^+^ homeostasis [4]. Another protective strategy is inactivation of the photosystem and cessation of energy transfer from phycobilisomes to PS II during the early stages of desiccation (to prevent photo-inhibition of photosynthetic apparatus) [4]. In fact, in *Ulva* sp., the existence of a PS I-driven cyclic electron flow has been shown to be an alternative pathway to eliminate harmful energy from the photosynthetic apparatus, contributing to the desiccation tolerance of these species [5]. Similarly, UV-absorbing substances, such as phenols and mycosporine-like amino acids, are significantly upregulated under UV-B exposure in the red seaweed *Neoporphyra haitanensis* [6].

In seaweeds, there are few reports about desiccation effects, physiological responses, and their influence on the geographic variation of species distribution. However, it has been reported that certain seaweed species from the *Pyropia*, *Porphyra*, and *Neoporphyra* genera [7,8,9,10,11] are generally highly tolerant to desiccation to prevent and/or reduce the cellular alteration induced under these conditions. In Chile, several *Porphyra* (*P. longissima* and *P. luchea*) and *Pyropia* (*P. orbicularis* and *P. variabilis*) species were recently recognized [12,13,14]. Both genera inhabit contrasting climatic areas along the Chilean coast between Arica (18° S) and South Patagonia (53° S) and show variable degrees of geographic range sizes or distributions along this coast [13], which could be explained by their contrasting tolerance limits to environmental stress.

The Chilean continental coastline is located in the southeastern Pacific and spans approximately 38 degrees of latitude (18°–56° S). It comprises three biogeographic provinces [15,16]: a northern zone, known as the “Peruvian Province” that spans from 18° to 30° S and includes temperate–warm biota, and a southern zone, known as the “Magellanic Province” that extends from 40°–42° to 56° S and possesses subantarctic-cold–temperate biota. Between these two zones is the “Intermediate Area” from 30°–40° to 42° S, which includes biota with mixed components of the two other major provinces. These three zones are separated mainly by two biogeographic coastal transition zones (CTZs), the first one located between 30° and 32° S and the second one located at approximately 42° S; both CTZs are characterized by breaks or discontinuities in the abundance of several species and by intra- and/or interspecific genetic differences for a subgroup of organisms, and they often coincide with the superposition in the distribution of several species from the adjoining zones. In seaweeds, the first CTZ represents a marked genetic break between two kelps: *Lessonia berteroana* from the Peruvian Province and *Lessonia spicata* from the Intermediate Area [17,18]. It has been demonstrated that *L. berteroana*, exposed to higher desiccation rates throughout its geographic range, is less susceptible to desiccation stress than *L. spicata*, both in its life cycle development and enzyme activity [19], which could explain its contrasted geographic distribution along the climatic gradient and CTZ characteristic of the Chilean coast. Thus, desiccation seems to be an important factor responsible for limiting the range of *Lessonia* species. Nonetheless, both *Lessonia* species thrive in the low intertidal zone; therefore, they are more susceptible to environmental factors that could increase desiccation intensity (e.g., air humidity, air and sea temperature, and irradiance [8,20]), in contrast to most of the desiccation-tolerant *Porphyra* and *Pyropia* species, which are able to grow in the high intertidal zone in the same geographic areas. Nonetheless, northern populations of upper intertidal seaweeds are likely more affected by drought and salinity stress during low tide due to the hyper-arid climate of northern Chile (18° S to 30° S), with high evaporation and lack of constant rains, compared with the wetter and lower evaporation conditions of more southern sites considered in this study (30° S to 41° S) [3].

On a global scale, *Porphyra* and *Pyropia* species are the marine crops of great importance (marketed as Nori or Laver), with a production of 2,810,600 tons in 2020 [21]. In Chile, the productive sector has relied mostly on the exploitation of natural algae populations (known as Luche), as its production is very small and mainly artisanal, with an annual value of 426 tons (dry biomass) in 2021 [22]. In particular, the upper intertidal specimens of *Pyropia orbicularis* have been shown to possess morphological, biochemical, and physiological adaptations to fully recover from >90% of water losses during a normal low tide cycle [7]. Indeed, *P. orbicularis* can be found in high abundance (in comparison with the other species) on flat rocky platforms during the summer, concurring with high temperature (max 40 °C), high radiation (maximum 3100 µmol photon m^−2^ s^−1^), and low humidity (minimum 15.9%) [23,24]. These adaptations include (i) antioxidant enzyme activities, such as ascorbate peroxidase, thioredoxin, catalase, and peroxiredoxin; (ii) attenuation of biomolecule oxidation; and (iii) metabolomic, proteomic, and transcriptomic changes involving several cellular pathways [7,8,19,20,24,25,26,27,28,29]. Thus, this species is considered a model of high stress tolerance, where several coordinated mechanisms are activated to cope with stressful environmental conditions.

Hence, desiccation could play an essential role in determining the high intertidal distribution of *Porphyra/Pyropia* species. In this context, considering the extended distribution of these species along the Chilean coast and the role of desiccation in determining the range limits of seaweed species, we hypothesize that tolerance to desiccation stress in *Porphyra/Pyropia* species is an intrinsic property of these uppermost intertidal species from both genera, independent of their latitudinal range of distribution. Therefore, the aim of this work was to explore the desiccation stress tolerance capacity in several Chilean *Porphyra/Pyropia* species at different latitudes, using antioxidant responses and oxidative stress markers.

## 2. Results

### 2.1. Specific Activity of Basal Metabolic and Antioxidant Enzymes and Metabolites along the Chilean Coast

**Figure 1:**
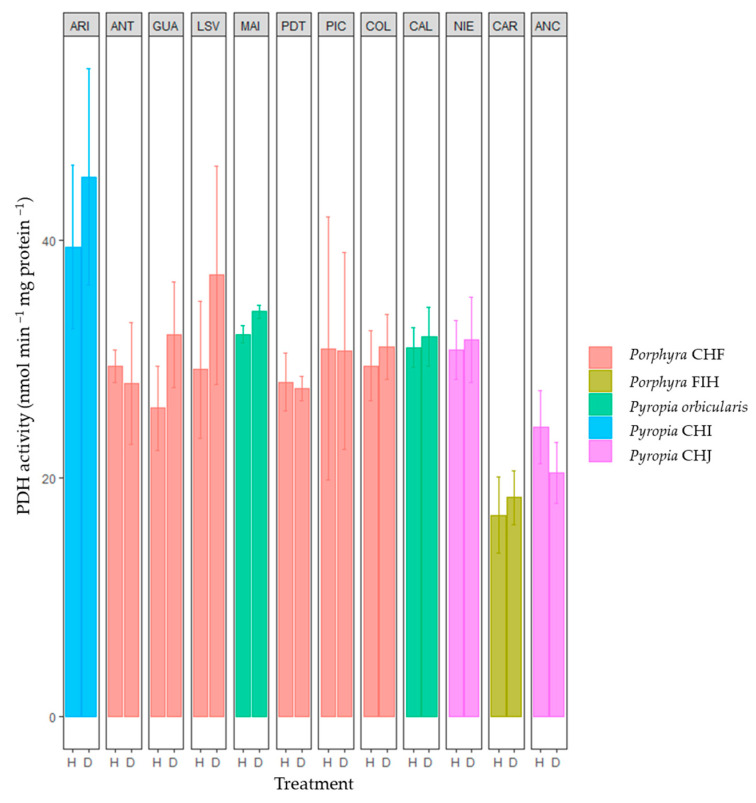
Specific activity of PDH (pyruvate dehydrogenase) during natural hydration (H) and desiccation (D) stress in the *Porphyra* and *Pyropia* species evaluated in this study. Values are the mean ± SD of three replicates. ARI, Arica; ANT, Antofagasta; GUA, Guanaquerillos; LSV, Los Vilos; MAI, Maitencillo; PDT, Punta de Tralca; PIC, Pichilemu; COL, Coliumo; CAL, Calfuco; NIE, Niebla; CAR, Carelmapu; and ANC, Ancud (see Table 1 and Figure 6 for in-depth information).

The results show a noticeable difference in the pyruvate dehydrogenase (PDH) activity among various *Porphyra* and *Pyropia* species and between sites (Figure 1). The results of Tukey’s test (Table S1) revealed that the main differences in PDH activity were the high values registered in some populations of *Pyropia* and *Porphyra* species from the northernmost sites (e.g., *Pyropia* CHI in Arica and *Porphyra* CHF in Los Vilos, respectively) and the relatively low values observed in some populations of *Pyropia* and *Porphyra* species from south Chile (e.g., *Porphyra* FIH in Carelmapu and *Pyropia* CHJ in Ancud).

**Figure 2:**
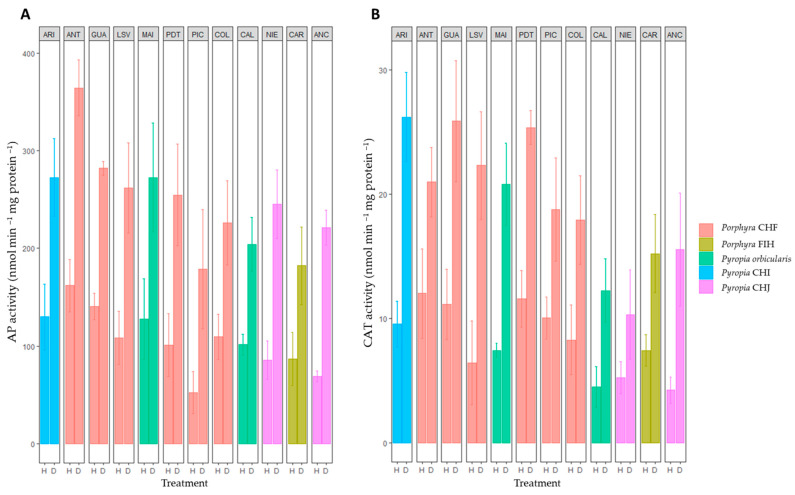
Specific activity of (**A**) AP (ascorbate peroxidase) and (**B**) CAT (catalase) during in situ hydration (H) and desiccation (D) stress in the *Porphyra* and *Pyropia* species evaluated in this study. Values are the mean ± SD of three replicates. ARI, Arica; ANT, Antofagasta; GUA, Guanaquerillos; LSV, Los Vilos; MAI, Maitencillo; PDT, Punta de Tralca; PIC, Pichilemu; COL, Coliumo; CAL, Calfuco; NIE, Niebla; CAR, Carelmapu; and ANC, Ancud (see Table 1 and Figure 6 for in-depth information).

**Figure 3:**
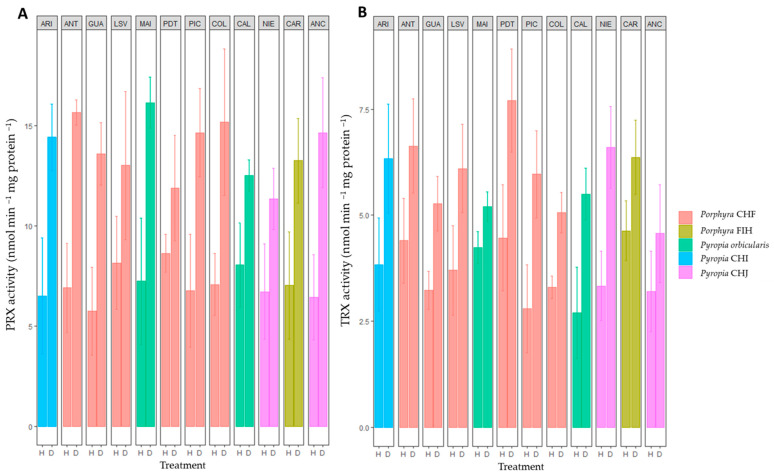
Specific activity of (**A**) PRX (peroxiredoxin) and (**B**) TRX (thioredoxin) during in situ hydration (H) and desiccation (D) stress in the *Porphyra* and *Pyropia* species evaluated in this study. Values are the mean ± SD of three replicates. ARI, Arica; ANT, Antofagasta; GUA, Guanaquerillos; LSV, Los Vilos; MAI, Maitencillo; PDT, Punta de Tralca; PIC, Pichilemu; COL, Coliumo; CAL, Calfuco; NIE, Niebla; CAR, Carelmapu; and ANC, Ancud (see Table 1 and Figure 6 for in-depth information).

In general, the activities of AP (Figure 2A), CAT (Figure 2B), PRX (Figure 3A), and TRX (Figure 3B) enzymes were significantly high in all *Porphyra*/*Pyropia* species at all the sites during natural desiccation when compared with natural hydration condition. A two-way ANOVA revealed a significant effect of species and site on the AP and CAT activities under both hydration and desiccation conditions, but no effect on PRX activity was registered (Table S2). A significant effect of site on TRX activity was also observed for desiccation; however, it remained non-significant under hydration conditions (Table S2).

Globally, the differences in activity can be explained mainly by the highest activity values recorded in *Porphyra* CHF for AP from Antofagasta (Figure 2A) and for CAT in *Pyropia* CHI from Arica and *Porphyra* CHF from Guanaquerillos (Figure 2B). Nonetheless, for AP and CAT, a gradual increase in the enzyme activities was generally observed within species from south to north (except within *Pyropia* CHJ for CAT). If we focus our attention on each species separately and on their mean PRX and TRX activities (Figure 3A,B), there was no distinct pattern of a latitudinal trend of higher or lower mean values (except within *Pyropia orbicularis* for PRX) but rather a plastic within species, locally dependent on the physiological performance.

**Figure 4:**
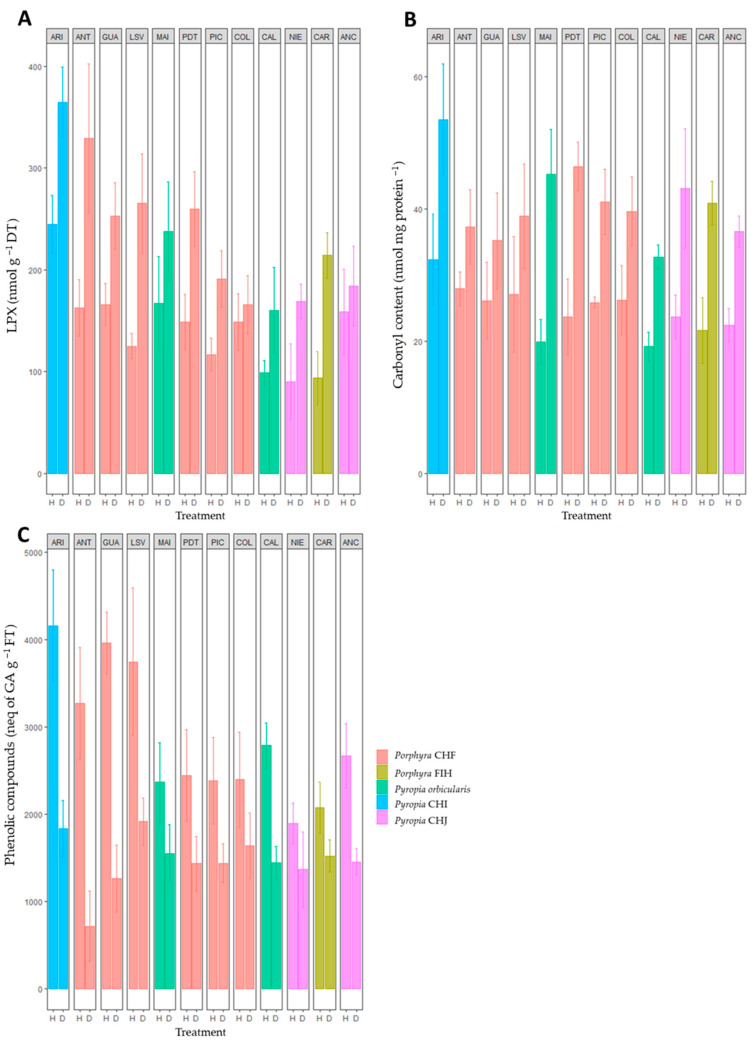
(**A**) Lipid peroxidation, (**B**) protein oxidation, and (**C**) phenolic compound concentrations during in situ hydration (H) and desiccation (D) stress in the *Porphyra* and *Pyropia* species evaluated in this study. Values are the mean ± SD of three replicates. ARI, Arica; ANT, Antofagasta; GUA, Guanaquerillos; LSV, Los Vilos; MAI, Maitencillo; PDT, Punta de Tralca; PIC, Pichilemu; COL, Coliumo; CAL, Calfuco; NIE, Niebla; CAR, Carelmapu; and ANC, Ancud (see Table 1 and Figure 6 for in-depth information).

Similar to antioxidant enzymes, the LPX and carbonyl contents were remarkably high in the desiccated population compared with the hydrated one (Figure 4A). A two-way ANOVA revealed a significant effect of species and site on LPX concentration during both naturally hydrated and desiccated conditions (Table S3 part A). On the contrary, only the species influenced the carbonyl levels (Figure 4B and Table S3 part A). Notably, *Pyropia* CHI, *Porphyra* CHF, and *Pyropia orbicularis* populations from the northern–central zone (e.g., Arica, Antofagasta, and Maitencillo) exhibited high LPX levels compared with southern populations (Figure 4A). However, only *Pyropia* CHI from the northern most site (Arica) exhibited the highest carbonyl level (Figure 4B).

The phenolic compounds displayed a significant decrease (50% on average) under natural desiccation compared with natural hydration (Figure 4C) in all *Pyropia* and *Porphyra* species, except from some southern populations. A two-way ANOVA showed a significant effect of site but not species on the phenolic compound levels during desiccation (Table S3 part A).

### 2.2. Differential Expression of Antioxidant Enzyme Transcripts

**Figure 5:**
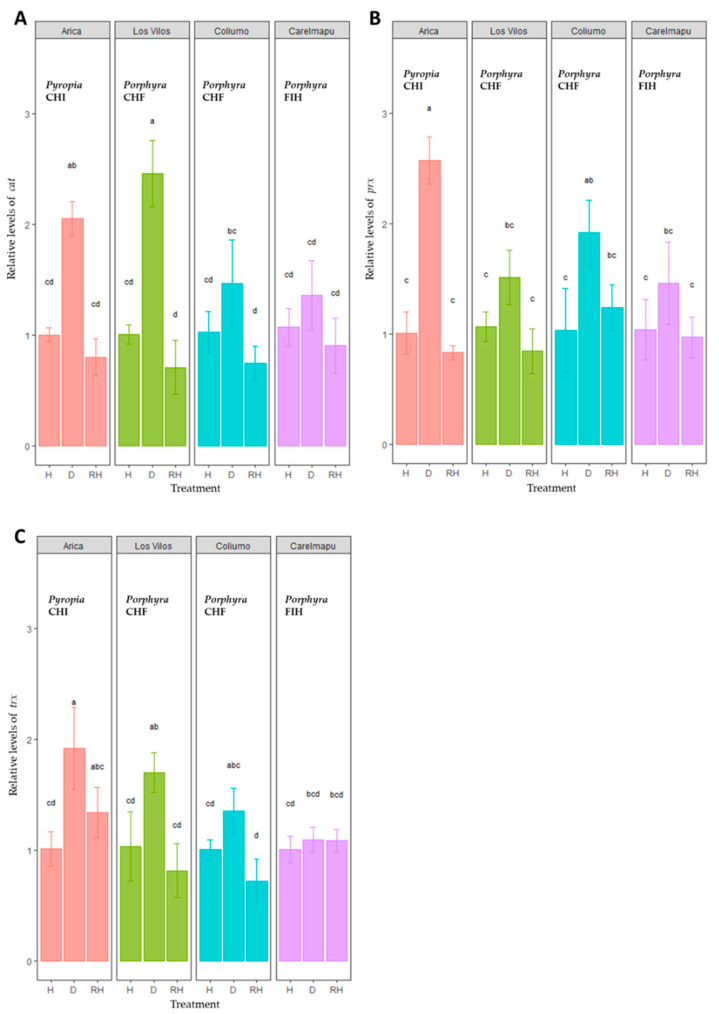
Relative expression level of (**A**) *cat*, (**B**) *prx*, and (**C**) *trx* transcripts during hydration (H), desiccation (D), and rehydration (RH) cycle in *Porphyra* and *Pyropia* species (see Table 1 and Figure 6 for details). Values are the mean ± SD of three replicates. The letters above the bar plots indicate the results of Tukey’s tests; means with the same letter are not significantly different at *p* ≥ 0.05. The colors of the histograms correspond to the sites evaluated.

In general terms, the transcript levels of *cat*, *prx*, and *trx* increased significantly during desiccation compared with hydration in the *Porphyra* and *Pyropia* species from the northernmost sites evaluated (i.e., Arica and Los Vilos) (Figure 5). A similar but smaller increase was observed in the *prx* transcripts of *Porphyra* CHF from Coliumo (Figure 5B). No differences were registered between hydration and desiccation in the *trx* transcripts from the southernmost sites (Figure 5C). A two-way ANOVA revealed a significant effect of treatment, site, and their interaction on the *cat*, *prx*, and *trx* expression (Table S4). In all cases, the expression levels during rehydration reached the same values registered during hydration (Figure 5), indicating a recovery of the transcript production to the basal levels. It is important to highlight that there was a clear pattern of high expression with a high activity of CAT, PRX, and TRX enzymes during desiccation (see Figure 2 and Figure 3). However, major analyses are required to evidence a significant relationship between the expression and enzymatic activity.

## 3. Discussion

We provide evidence of a significant activation of antioxidant enzymes and transcripts (principally *cat* and *prx*) and the participation of phenolic antioxidant compounds as generalized mechanisms of desiccation tolerance in *Porphyra* and *Pyropia* species along the Chilean coastal zone. In addition, the levels of stress response were generally dependent on the latitudinal distribution, with more pronounced tolerance responses in northern populations. The results obtained validate our predictions, as the desiccation stress tolerance in *Porphyra*/*Pyropia* species is an intrinsic property of these uppermost intertidal species from both genera. Independent of their latitudinal range of distribution along the Chilean coast, the northern species and populations undergo harsher regimes of environmental stresses than central and southern ones. Indeed, if we consider all species and populations of *Porphyra* and *Pyropia* as a whole, the lipid and protein oxidation damage (i.e., LPX and carbonyl) levels increase gradually from south to north along the Chilean coast (mainly during desiccation), which was clearly evident with a higher LPX accumulation than the carbonyl levels. To combat the oxidative damage, a gradual increase in the antioxidant enzymes, both at the biochemical and transcript level, was noticed in the populations from south to north during the desiccation cycle. Specifically, the relative expression of genes, including *cat*, *prx*, and *trx*, were significantly higher in the northern species and populations compared with the central and southern ones during desiccation. Indeed, previously we demonstrated that the transcriptional modulation of the desiccation tolerance factors in *P. orbicularis* explains its successful recuperation after a water deficit [29]. Moreover, the four northernmost populations studied in this work showed a higher concentration of phenolic compounds during hydration (than did the southern ones), which were significantly reduced during desiccation, likely to cope with the desiccation and other stresses (e.g., salinity and UV radiation) at low tide [30]. Thus, differential responses to air exposure clarify the seaweed distribution along the intertidal rocky zones in a broad range of latitudinal distributions.

In natural habitats, desiccation stress often overlaps with salinity and high light stress. Therefore, differences between species in the extent of their geographical and/or (local) microhabitat distributions could arise from their differential capacities to tolerate other important environmental constraints [31]. One of the main environmental restrictions to thrive in the high intertidal in marine organisms is the necessity to adapt to a wide range of salinities and desiccation stress regimes and, therefore, to spend a large amount of their metabolic energy budget on the process of osmotic adjustment [32]. Likewise, some high intertidal algae usually possess a relatively desiccation- and salt-insensitive photosynthesis and respiration [33]. Salinity-tolerant seaweed species maintain osmotic homeostasis using inorganic ions and small organic osmolytes. Indeed, the type and concentration of the osmolytes have been shown to vary geographically and seasonally among algal taxa. For example, three different species of *Porphyra* species from Australia, the North Sea, and the Pacific Coast of the USA showed different types and variable ratios of the low-molecular-weight carbohydrates (i.e., osmolytes) L-isofloridoside, floridoside, and D-isofloridoside [34]. Recently, [35] demonstrated considerable changes in floridosides in both *Neoporphyra haitanensis* thalli and conchocelis under salt stress. In the case of the *Porphyra* and *Pyropia* species from the coast of Chile, whose wide range of tolerance to oxidative stress was shown in this study, the variability in the extent of their geographic and/or microhabitat distributions could proceed from their differential capacities to maintain osmotic homeostasis ensuring the protection of biological molecules using osmolytes during low tide and/or extreme climatic events. For example, this could explain the splitting of the recently diverging species *Pyropia variabilis* (along the northern coast), *Pyropia orbicularis*, and *Pyropia* CHJ (both along the central and southern coasts) [13,14]. These three species span across the three biogeographic provinces with contrasting thermohaline variability and disparate river flow and precipitation regimes.

For the first time, this study integrated the biochemical and genetic responses of diverse *Porphyra*/*Pyropia* species to better understand their physiological dynamics of tolerance over a wide latitudinal range. This confirms the high tolerance of theses genera to the environmental desiccation stress and their high distribution in higher intertidal zones. In spite of the high desiccation tolerance of all of the *Porphyra* and *Pyropia* species shown in this study, a higher sampling effort per site and wide geographical extent would be required to improve the characterization of the within-species variation patterns concerning their physiological tolerance responses to desiccation stress, except for *Porphyra* CHF, which had a sufficient sampling effort and optimal representation along its geographic distribution. In addition, major analyses are required to understand the low participation during the desiccation stress of pyruvate dehydrogenase (PDH) in these species because of their important role in the basal metabolism. In fact, it was previously demonstrated there were no changes in the PDH activity in *P. orbicularis* during stress [29], and contrary high activity was determined during rehydration. PDH is an essential enzymatic complex that produces energy (ATP and GTP), NADH, and FADH_2_, thus facilitating the biosynthesis of complex molecules during stressful conditions and subsequent recuperation [29]. In plants, PDH inhibition has been related to both high light and NDPH concentration [36,37]. Therefore, similar mechanisms will be generated during environmental stress in *Porphyra* and *Pyropia* genera.

In recent years, several studies on these genera have provided insight into the tolerance mechanisms underlying their distribution in highly stressful environments, e.g., [38,39,40,41,42,43]. For example, *Porphyra umbilicalis* exhibits increased antioxidant metabolism, which could contribute to its success in colonizing stressful habitats [38]. In *Pyropia yezoensis*, the *HSP70* (heat shock protein) gene family showed upregulated expression under different degrees of desiccation stress [43]. In addition, the description of the life cycle of several species in both natural and controlled [44,45] environmental conditions and the high activation of the physiological tolerance responses compared with the species of other genera [8,20,27] allow for positioning of the *Porphyra* and *Pyropia* complexes as important models for studies not only at the ecological but also at the biotechnological level.

The acquisition of physiological tolerance traits in this group of algae has been scarcely studied worldwide [46]. Interesting aspects are required today to understand this exceptional and diverse algal complex. As this group of algae is an important part of the world’s food supply, further studies are required at the biochemical and molecular levels, for example, to stimulate the production of nutritional or functional compounds and to improve the dietary quality [47,48]. Therefore, further studies associated with both aquaculture and biomass quality of *Porphyra* and *Pyropia* are required.

## 4. Materials and Methods

### 4.1. Sampling, Study Sites, and Experimental Design

**Figure 6:**
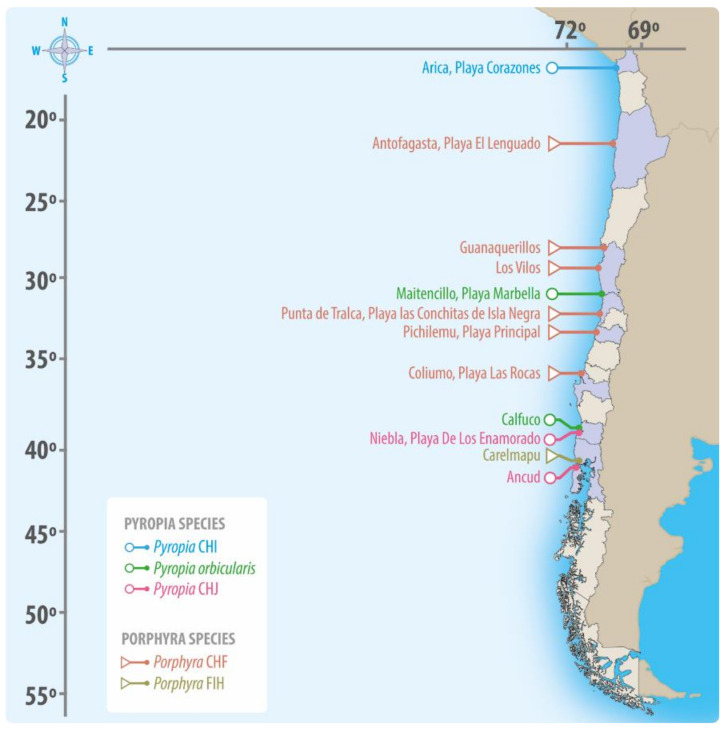
Sampling sites along the Chilean coast of the *Porphyra* and *Pyropia* species studied in this work.

To evaluate the physiological responses of *Porphyra* and *Pyropia* species to desiccation stress, samples were collected from the uppermost intertidal zone of 12 different locations along the Chilean coast (Table 1 and Figure 6).

For in situ determination of the oxidative stress, vegetative individuals of *Porphyra* and *Pyropia* species were collected along 100–150 m coastal transects during high tide (naturally hydrated, 100% of relative water content) and under 4 h of low tide (naturally desiccated, 90–96% loss of relative water content). In both cases, the samples were immediately frozen in liquid nitrogen until further biochemical analysis. For the in vitro desiccation experiments concerning antioxidant (*cat*, *prx*, and *trx*) transcripts, hydrated vegetative individuals of *Porphyra* and *Pyropia* species from four sites (Arica, Los Vilos, Coliumo, and Carelmapu) before collection were kept in plastic bags with seawater at 5–7 °C and transported to the seaweed laboratory of the Universidad Andrés Bello. The fronds were carefully and exhaustively rinsed with 0.45 µm filtered seawater and acclimated in filtered seawater during 12 h in a culture chamber at 12–14 °C, under 70–80 µm photon m^−2^ s^−1^ irradiance. Subsequently, a set of fronds was blotted dry and exposed to desiccation in a growth chamber at 14 °C and 70–80 µmol photon m^−2^ s^−1^ irradiance for 4 h. Finally, a subset of fronds dehydrated for 4 h was immediately re-hydrated for 2 h with 0.45 µm filtered seawater to characterize the recovery to the oxidative stress induced by desiccation.

### 4.2. Extraction and Quantification of Protein Extracts

Algal samples were frozen in liquid nitrogen and homogenized in a mortar with a pestle. Proteins were precipitated with ammonium sulfate, stabilized in 2 mM 2-mercaptoethanol [49], and quantified using a Thermo Scientific Pierce BCA Protein Assay Kit (Thermo Fisher Scientific, Rockford, Illinois, USA) on the basis of the bicinchoninic acid method [50].

### 4.3. Biochemical Determinations

The specific activity of the antioxidant enzymes ascorbate peroxidase (AP) and catalase (CAT) was determined according to [49]. Peroxiredoxin activity (PRX) was determined according to [51], and pyruvate dehydrogenase (PDH) and thioredoxin (TRX) according to [29]. Phenolic compounds were determined using Folin–Ciocalteau reagent according to [49] and expressed as nanoequivalents of gallic acid using a calibration curve prepared with 10–50 nmoles of gallic acid.

The lipid peroxidation levels were determined as the amount of thiobarbituric acid reactive species (i.e., lipoperoxides) as previously described [49]. The protein oxidation levels were determined on the basis of the reaction of carbonyls with 2,4-dinitrophenyl hydrazine (DNPH) [7]. The carbonyl concentration was calculated using the molar extinction coefficient of the carbonyl–DNPH complex (ε = 22 mM^−1^ cm^−1^) and expressed as nmol of DNPH incorporated per milligram of protein.

### 4.4. Differential Gene Expression

Differential gene expression was determined in four populations distributed along the Chilean coast: Arica (*Pyropia* CHI), Los Vilos (*Porphyra* CHF), Coliumo (*Porphyra* CHF), and Carelmapu (*Porphyra* FIH) (Table 1) under in vitro desiccation and rehydration conditions. The hydration conditions were those from natural high tide. The following genes were analyzed: *cat* (catalase), *prx* (peroxiredoxin), *trx* (thioredoxin), and the internal reference gene *sen* (senescence-associated protein) as the housekeeping gene (Supplementary Materials Table S5).

Total RNA was isolated from 1–1.5 g of fronds. Tissue, frozen with liquid nitrogen, was homogenized in 5 mL of lysis buffer containing 4 M guanidinium thiocyanate, 25 mM EDTA, 200 mM sodium acetate, 2% polyvinylpyrrolidone (PVP-40), and 1% 2-mercaptoethanol. The homogenate was incubated for 10 min at 70 °C, under constant agitation, in the presence of 20% sarcosine, and then centrifuged for 5 min at 16,000× *g*. The RNA present in the supernatant solution was purified and re-extracted using a RNeasy Mini Kit (Qiagen, Hilden, Germany) according to the manufacturer’s protocol. The remaining DNA was removed using DNase I Amplification Grade (Invitrogen). The RNA quality and yield were assessed by spectrophotometry (NanoDropTM 1000 Spectrophotometer, Thermo Scientific, Wilmington, DE, USA) and denaturing 1.2% agarose gel electrophoresis.

RNA was reverse-transcribed using the Superscript II reverse transcriptase (Thermo Fisher Scientific, Carlsbad, CA, USA). First, 5 μM of Oligo(dT) 12–18 was added to 3–5 μg of total RNA and annealed at 65 °C for 5 min. Then, 4 μL of First Strand Buffer, 2 μL of 0.1M DTT, and 200 U Superscript II reverse transcriptase were added and incubated at 42 °C for 50 min. Incubation of the reaction was stopped at 70 °C for 15 min. The cDNA samples obtained were analyzed spectrophotometrically and stored at –80 °C until use.

For each real-time PCR, 200 ng of template cDNA was mixed with 5 μL of 2 × SYBR Green PCR master mix and 300–600 nM of each of the forward and reverse primers (Supplementary Materials Table S5) to a final volume of 10 μL. For amplification, the program used was a first step at 90 °C for 10 s; 40 cycles of activation at 90 °C for 3 s and annealing extension at 60 °C for 30 s, and a thermal denaturing step to generate the dissociation curves to verify amplification specificity. All reactions were performed in triplicate, and the Pfaffl method (ΔCt) was used for expression analysis relative to the *sen* gene [52].

### 4.5. Statistical Analysis

Significant differences between sites and species were determined using two-way analysis of variance (ANOVA) when comparing the hydrated and desiccated treatments for the biochemistry analyses. The same analysis was performed for the significant differences between sites and treatments in the differential gene expression assays. Posteriori analysis of Tukey’s multiple comparisons test (T) was performed to detect differences at each condition. The level of significance for all statistical tests was *p* < 0.05.

## Figures and Tables

**Table 1:** Study sites indicating the *Porphyra* and *Pyropia* species determined by morphological and molecular analysis according to [12,13,14].

Site Name	Geographic Coordinates	Species
Arica, Playa Corazones (ARI)	18°32′55″ S, 70°19′52″ W	*Pyropia* CHI
Antofagasta, Playa El Lenguado (ANT)	23°46′30″ S, 70°28′33″ W	*Porphyra* CHF
Guanaquerillos (GUA)	30°11′41″ S, 71°25′18″ W	*Porphyra* CHF
Los Vilos (LSV)	31°55′10″ S, 71°31′08″ W	*Porphyra* CHF
Maitencillo, Playa Marbella (MAI)	32°39′13″ S, 71°26′37″ W	*Pyropia orbicularis*
Punta de Tralca, Playa las Conchitas de Isla Negra (PDT)	33°26′22″ S, 71°41′18″ W	*Porphyra* CHF
Pichilemu, Playa Principal (PIC)	34°22′50″ S, 72°00′58″ W	*Porphyra* CHF
Coliumo, Playa Las Rocas (COL)	36°31′36″ S, 72°57′23″ W	*Porphyra* CHF
Calfuco (CAL)	39°46′48″ S, 73°23′34″ W	*Pyropia orbicularis*
Niebla, Playa De Los Enamorados (NIE)	39°51′36″ S, 73°23′38″ W	*Pyropia* CHJ
Carelmapu (CAR)	41°44′32″ S, 73°44′36″ W	*Porphyra* FIH
Ancud, Playa Mar Brava (ANC)	41°51′55″ S, 74°00′45″ W	*Pyropia* CHJ

## Data Availability

Derived data supporting the findings of this study are available from the corresponding author (L. Contreras-Porcia) on request.

## Supplementary Materials

The following supporting information can be downloaded at: https://www.mdpi.com/article/10.3390/plants12010012/s1, Table S1: (A) Two-way ANOVA carried out comparing the effects of species and sites on pyruvate dehydrogenase (PDH) levels exposed to desiccation and hydration treatments. *p*-values lower than 0.05 indicate significant differences. Df: degrees of freedom; Sum q: Sum square; Mean Sq: Mean square; F: *f*-value; and Pr(>F): *p* values. (B) Tukey’s test carried out comparing different species exposed to desiccation and hydration treatments, Table S2: (A) Two-way ANOVA carried out comparing the effects of species and sites on ascorbate peroxidase (AP), catalase (CAT), peroxiredoxin (PRX), and thioredoxin (TRX) levels exposed to desiccation and hydration treatments. *p*-values lower than 0.05 indicate significant differences. Df: degrees of freedom; Sum q: Sum square; Mean Sq: Mean square; F: *f*-value; and Pr(>F): *p*-values. (B) Tukey’s test carried out comparing different species exposed to desiccation and hydration treatments, Table S3: (A) Two-way ANOVA carried out comparing the effects of species and sites on lipoperoxides (LPX), carbonyls (CAR), and phenolic compounds levels exposed to desiccation and hydration treatments. *p*-values lower than 0.05 indicate significant differences. Df: degrees of freedom; Sum q: Sum square; Mean Sq: Mean square; F: *f*-value; and Pr(>F): *p*-values. (B) Tukey’s test carried out comparing different species exposed to desiccation and hydration treatments, Table S4: Two-way ANOVA carried out comparing the effects of treatments (hydration, desiccation, and rehydration) and sites on gene expression in vitro of catalase (*cat*), peroxiredoxin (*prx*), and thioredoxin (*trx*) levels for *Porphyra* and *Pyropia* species. *p*-values lower than 0.05 indicate significant differences. Df: degrees of freedom; Sum q: Sum square; Mean Sq: Mean square; F: *f*-value; and Pr(>F): *p*-values, Table S5: Primers sequences used for real-time PCR experiments. Source: [25,29].
