# Supplementary material for: Desiccation Stress Tolerance in Porphyra and Pyropia Species: A Latitudinal Analysis along the Chilean Coast
Source: Plants (Basel). 2022 Dec 20;12(1):12. doi: 10.3390/plants12010012 (PMC9824847; doi:10.3390/plants12010012)
Supplement: Supplementary file 1 [file plants-12-00012-s001.zip › plants-2095258-supplementary.pdf]

plants

MDPI

Supplementary material

Desiccation Stress Tolerance in *Porphyra* and *Pyropia* Species: A Latitudinal Analysis along the Chilean Coast

Loretto Contreras-Porcia [1,2,3,4,*], Andrés Meynard [1,2,3,4], Florentina Piña [1,2,3,4], Manoj Kumar [5], Carlos Lovazzano [6,7], Alejandra Núñez [1,2,3,4] and María Rosa Flores-Molina [1,2,3,4]

1 Departamento de Ecología y Biodiversidad, Facultad de Ciencias de la Vida, Universidad Andres Bello, República 440, Santiago 8370251, Chile
2 Centro de Investigación Marina Quintay (CIMARQ), Facultad de Ciencias de la Vida, Universidad Andres Bello, Quintay 2531015, Chile
3 Center of Applied Ecology and Sustainability (CAPES), Santiago 8331150, Chile
4 Instituto Milenio en Socio-Ecología Costera (SECOS), Santiago 8370251, Chile
5 Climate Change Cluster, Faculty of Science, University of Technology Sydney, Sydney, NSW 2007, Australia
6 Instituto de Bioquímica y Microbiología, Facultad de Ciencias, Universidad Austral de Chile, Valdivia 5110566, Chile
7 Millennium Institute for Integrative Biology (iBio), Santiago 8331150, Chile
* Correspondence: lorettocontreras@unab.cl

Table S1. A) Two-way ANOVA carried out comparing the effects of species and sites on pyruvate dehydrogenase (PDH) levels exposed to desiccation and hydration treatments. *P* values lower than *0.05* indicate significant differences. Df: degrees of freedom; Sum q: Sum square; Mean Sq: Mean square; F: *f* value; Pr(>F): *P* values. B) Tukey test carried out comparing different species exposed to desiccation and hydration treatments.

A) Two-way ANOVA:

Desiccation

	Df	Sum Sq	Mean Sq	F value	Pr (>F)	
**Species**	4	1255.29	313.823	11.7804	1.951e-05	***
**Sites**	7	372.72	53.246	1.9988	0.09747	
**Residuals**	24	639.35	26.639			

Hydration

	Df	Sum Sq	Mean Sq	F value	Pr (>F)	
**Species**	4	815.03	203.758	9.3714	0.000104	***
**Sites**	7	107.20	15.314	0.7043	0.668420	
**Residuals**	24	521.82	21.743			

B) Tukey test:

Desiccation

Species	diff	lwr	upr	p adj

***Po*FIH-*Po*CHF**	-12667778	-22150038	-3.185518	0.0050951
***Py.orbicularis-Po*CHF**	1.875556	-5.292359	9.043470	0.9365255
***Py*CHI-*Po*CHF**	14.245556	4.763296	23.727815	0.0015315
***Py*CHJ-*Po*CHF**	-4.989444	-12.157359	2.178470	0.2734635
***Py.orbicularis-Po*FIH**	14.543333	3.791461	25.295205	0.0045221
***Py*CHI-*Po*FIH**	26.913333	14.498141	39.328526	0.0000123
***Py*CHJ-*Po*FIH**	7.678333	-3.073539	18.430205	0.2509769
Py*CHI-*Py.orbicularis	12.370000	1.618128	23.121872	0.0185978
Py*CHJ-*Py.orbicularis	-6.865000	-15.643867	1.913867	0.1782825
***Py*CHJ-*Py*CHI**	-19.235000	-29.986872	-8.483128	0.0001879

Hydration

**Species**	**diff**	**lwr**	**upr**	**p adj**
***Po*FIH-*Po*CHF**	-11.886111	-20.4526574	-3.319565	0.0035206
***Py.orbicularis-Po*CHF**	2.715556	-3.7601447	9.191256	0.7313290
***Py*CHI-*Po*CHF**	10.613889	2.0473426	19.180435	0.0101071
***Py*CHJ-*Po*CHF**	-1.267778	-7.7434781	5.207923	0.9772522
***Py.orbicularis-Po*FIH**	14.601667	4.8881162	24.315217	0.0015216
***Py*CHI-*Po*FIH**	22.500000	11.2837581	33.716242	0.0000389
***Py*CHJ-*Po*FIH**	10.61833	0.9047829	20.331884	0.0273324
Py*CHI-*Py.orbicularis	7.898333	-1.8152171	17.611884	0.1509302
Py*CHJ-*Py.orbicularis	-3.983333	-11.9144141	3.947747	0.5848039
***Py*CHJ-*Py*CHI**	-11.881667	-21.5952171	-2.168116	0.0112824

Table S2. A) Two-way ANOVA carried out comparing the effects of species and sites on ascorbate peroxidase (AP), catalase (CAT), peroxiredoxin (PRX) and thioredoxin (TRX) levels exposed to desiccation and hydration treatments. *P* values lower than *0.05* indicate significant differences. Df: degrees of freedom; Sum q: Sum square; Mean Sq: Mean square; F: *f* value; Pr(>F): *P* values. B) Tukey test carried out comparing different species exposed to desiccation and hydration treatments.

AP

Desiccation

	**Df**	**Sum Sq**	**Mean Sq**	**F value**	**Pr (>F)**	
**Species**	4	19849	4962.2	2.9887	0.0390446	*

**Sites**	7	65402	9343.2	5.6273	0.0006247	***
**Residuals**	24	39848	1660.3			

Hydration

	**Df**	**Sum Sq**	**Mean Sq**	**F value**	**Pr (>F)**	
**Species**	4	8847.2	2211.8	3.3981	0.024478	*
**Sites**	7	22389.9	3198.6	4.9142	0.001488	**
**Residuals**	24	15621.2	650.9			

CAT

Desiccation

	**Df**	**Sum Sq**	**Mean Sq**	**F value**	**Pr (>F)**	
**Species**	4	602.18	150.545	11.5392	2.285e-05	***
**Sites**	7	314.81	44.973	3.4472	0.01073	*
**Residuals**	24	313.11	13.046			

Hydration

	**Df**	**Sum Sq**	**Mean Sq**	**F value**	**Pr (>F)**	
**Species**	4	162.118	40.530	8.2268	0.0002514	***
**Sites**	7	85.179	12.168	2.4700	0.0464446	*
**Residuals**	24	118.236	4.927			

PRX

Desiccation

	**Df**	**Sum Sq**	**Mean Sq**	**F value**	**Pr (>F)**
**Species**	4	8.218	2.0546	0.4039	0.8039
**Sites**	7	66.688	9.5269	1.8727	0.1191
**Residuals**	24	122.096	5.0873		

Hydration

	**Df**	**Sum Sq**	**Mean Sq**	**F value**	**Pr (>F)**
**Species**	4	4.685	1.1713	0.2108	0.9299
**Sites**	7	17.089	2.4412	0.4394	0.8674
**Residuals**	24	133.326	5.5552		

TRX

Desiccation

	Df	Sum Sq	Mean Sq	F value	Pr (>F)	
**Species**	4	4.4597	1.11493	1.2547	0.31498	
**Sites**	7	20.2011	2.88587	3.2476	0.01433	*
**Residuals**	24	21.3267	0.88861			

Hydration

	Df	Sum Sq	Mean Sq	F value	Pr (>F)
**Species**	4	4.0847	1.02118	1.2751	0.3073
**Sites**	7	10.3050	1.47214	1.8383	0.1258
**Residuals**	24	19.2200	0.80083		

B) Tukey test:

AP

Desiccation

Species	diff	lwr	upr	p adj
***Po*FIH-*Po*CHF**	-79.16667	-154.026477	-4.306857	0.0345492
***Py.orbicularis*-*Po*CHF**	-23.01667	-79.605364	33.572031	0.7523027
***Py*CHI-*Po*CHF**	11.31000	-63.549810	86.169810	0.9913402
***Py*CHJ-*Po*CHF**	-27.97667	-84.565364	28.612031	0.5989888
***Py.orbicularis*-*Po*FIH**	56.15000	-28.733046	141.033046	0.3201922
***Py*CHI-*Po*FIH**	90.47667	-7.537832	188.491166	0.0803454
***Py*CHJ-*Po*FIH**	51.19000	-33.693046	136.073046	0.4093889
Py*CHI-*Py.orbicularis	34.32667	-50556379	119.209713	0.7561282
Py*CHJ-*Py.orbicularis	-4.96000	-74.266717	64.346717	0.9995266
***Py*CHJ-*Py*CHI**	-39.28667	-124.169713	45.596379	0.6556856

Hydration

Species	diff	lwr	upr	p adj
***Po*FIH-*Po*CHF**	-25.398333	-72.26905	21.4723840	0.5137661
***Py.orbicularis*-*Po*CHF**	2.183333	-33.24760	37.6142653	0.9997381
***Py*CHI-*Po*CHF**	17.461667	-29.40905	64.3323840	0.8060563
***Py*CHJ-*Po*CHF**	-34.918333	-70.34927	0.5125986	0.0547668
***Py.orbicularis*-*Po*FIH**	27.581667	-25.56473	80.7280646	0.5546557
***Py*CHI-*Po*FIH**	42.860000	-18.50817	104.2281743	0.2704911
***Py*CHJ-*Po*FIH**	-9.520000	-62.66640	43.6263979	0.9835987

Py*CHI-*Py.orbicularis	15.278333	-37.86806	68.4247313	0.9130408
Py*CHJ-*Py.orbicularis	-37.101667	-80.49552	6.2921855	0.1195949
***Py*CHJ-*Py*CHI**	-52.380000	-105.52640	0.7663979	0.0547506

CAT

Desiccation

Species	diff	lwr	upr	p adj
***Po*FIH-*Po*CHF**	-6.653333	-13.289143	-0.0175234	0.0491716
***Py.orbicularis*-*Po*CHF**	-5.351667	-10.367867	-0.3354659	0.0325016
***Py*CHI-*Po*CHF**	4.343333	-2.292477	10.9791433	0.3301783
***Py*CHJ-*Po*CHF**	-8.940000	-13.956201	-3.9237992	0.0001975
***Py.orbicularis*-*Po*FIH**	1.301667	-6.222635	8.8259679	0.9855908
***Py*CHI-*Po*FIH**	10.996667	2.308352	19.6849813	0.0083829
***Py*CHJ-*Po*FIH**	-2.286667	-9.810968	5.2376346	0.8958248
Py*CHI-*Py.orbicularis	9.695000	2.170699	17.2193012	0.0071377
Py*CHJ-*Py.orbicularis	-3.588333	-9.731900	2.5552329	0.4407847
***Py*CHJ-*Py*CHI**	-13.283333	-20.807635	-5.7590321	0.0002233

Hydration

Species	diff	lwr	upr	p adj
***Po*FIH-*Po*CHF**	-2.4822222	-6.559958	1.5955139	0.4002365
***Py.orbicularis*-*Po*CHF**	-3.9405556	-7.023034	-0.8580768	0.0076664
***Py*CHI-*Po*CHF**	-0.3622222	-4.439958	3.7155139	0.9988908
***Py*CHJ-*Po*CHF**	-5.1855556	-8.268034	-2.1030768	0.0004105
***Py.orbicularis*-*Po*FIH**	-1.4583333	-6.082051	3.1653848	0.8827424
***Py*CHI-*Po*FIH**	2.1200000	-3.219010	7.4590098	0.7679831
***Py*CHJ-*Po*FIH**	-2.7033333	-7.327051	1.9203848	0.4397992
Py*CHI-*Py.orbicularis	3.5783333	-1.045385	8.2020515	0.1859837
Py*CHJ-*Py.orbicularis	-1.2450000	-5.020250	2.5302500	0.8652242
***Py*CHJ-*Py*CHI**	-4.8233333	-9.447051	-0.1996152	0.0379291

PRX

Desiccation

Species	diff	lwr	upr	p adj

***Po*FIH-*Po*CHF**	-0.7500000	-4.893764	3.393764	0.9829565
***Py.orbicularis*-*Po*CHF**	0.3350000	-2.797391	3.467391	0.9977078
***Py*CHI-*Po*CHF**	0.4200000	-3.723764	4.563764	0.9981407
***Py*CHJ-*Po*CHF**	-1.0016667	-4.134057	2.130724	0.8775470
***Py.orbicularis*-*Po*FIH**	1.0850000	-3.613586	5.783586	0.9588348
***Py*CHI-*Po*FIH**	1.1700000	-4.255460	6.595460	0.9677217
***Py*CHJ-*Po*FIH**	-0.2516667	-4.950253	4.446920	0.9998499
Py*CHI-*Py.orbicularis	0.0850000	-4.613586	4.783586	0.9999980
Py*CHJ-*Py.orbicularis	-1.3366667	-5.173046	2.499713	0.8406764
***Py*CHJ-*Py*CHI**	-1.4216667	-6.120253	3.276920	0.8972846

Hydration

Species	diff	lwr	upr	p adj
***Po*FIH-*Po*CHF**	-0.1944444	-4.524575	4.135686	0.9999255
***Py.orbicularis*-*Po*CHF**	0.4222222	-2.851049	3.695493	0.9952652
***Py*CHI-*Po*CHF**	-0.7144444	-5.044575	3.615686	0.9879289
***Py*CHJ-*Po*CHF**	-0.6344444	-3.907716	2.638827	0.9780692
***Py.orbicularis*-*Po*FIH**	0.6166667	-4.293240	5.526573	0.9957271
***Py*CHI-*Po*FIH**	-0.5200000	-6.189472	5.149472	0.9987424
***Py*CHJ-*Po*FIH**	-0.4400000	-5.349907	4.469907	0.9988518
Py*CHI-*Py.orbicularis	-1.1366667	-6.046573	3.773240	0.9584666
Py*CHJ-*Py.orbicularis	-1.0566667	-5.065589	2.952255	0.9349305
***Py*CHJ-*Py*CHI**	0.0800000	-4.829907	4.989907	0.9999987

TRX

Desiccation

Species	diff	lwr	upr	p adj
***Po*FIH-*Po*CHF**	0.24444444	-1.4873870	1.9762759	0.9933140
***Py.orbicularis*-*Po*CHF**	-0.77222222	-2.0813637	0.5369193	0.4311017
***Py*CHI-*Po*CHF**	0.21111111	-1.5207203	1.9429425	0.9961922
***Py*CHJ-*Po*CHF**	-0.53888889	-1.8480304	0.7702526	0.7442177
***Py.orbicularis*-*Po*FIH**	-1.01666667	2.9803789	0.9470456	0.5568996
***Py*CHI-*Po*FIH**	-0.03333333	-2.3008329	2.2341663	0.9999991
***Py*CHJ-*Po*FIH**	-0.78333333	-2.7470456	1.1803789	0.7650446

Py*CHI-*Py.orbicularis	0.98333333	-0.9803789	2.9470456	0.5874958
Py*CHJ-*Py.orbicularis	0.23333333	-1.3700310	1.8366977	0.9924885
***Py*CHJ-*Py*CHI**	-0.75000000	-2.7137123	1.2137123	0.7918131

Hydration

Species	diff	lwr	upr	p adj
***Po*FIH-*Po*CHF**	0.9833333	-0.6607386	2.6274053	0.4174842
***Py.orbicularis*-*Po*CHF**	-0.1833333	-1.4261349	1.0594682	0.9920906
***Py*CHI-*Po*CHF**	0.1833333	-1.4607386	1.8274053	0.9973033
***Py*CHJ-*Po*CHF**	-0.3833333	-1.6261349	0.8594682	0.8907612
***Py.orbicularis*-*Po*FIH**	-1.1666667	-3.0308690	0.6975357	0.3732190
***Py*CHI-*Po*FIH**	-0.8000000	-2.9525955	1.3525955	0.8074087
***Py*CHJ-*Po*FIH**	-1.3666667	-3.2308690	0.4975357	0.2287860
Py*CHI-*Py.orbicularis	0.3666667	-1.4975357	2.2308690	0.9768621
Py*CHJ-*Py.orbicularis	-0.2000000	-1.7221149	1.3221149	0.9949168
***Py*CHJ-*Py*CHI**	0.5666667	-2.4308690	1.2975357	0.8957494

Table S3. A) Two-way ANOVA carried out comparing the effects of species and sites on lipoperoxides (LPX), carbonyls (CAR) and phenolic compounds levels exposed to desiccation and hydration treatments. *P* values lower than *0.05* indicate significant differences. Df: degrees of freedom; Sum q: Sum square; Mean Sq: Mean square; F: *f* value; Pr(>F): *P* values. B) Tukey test carried out comparing different species exposed to desiccation and hydration treatments.

A) LPX

Desiccation

	Df	Sum Sq	Mean Sq	F value	Pr (>F)	
**Species**	4	81043	20260.7	12.6014	1.158e-05	***
**Sites**	7	59843	8548.9	5.3171	0.0009049	***
**Residuals**	24	38588	1607.8			

Hydration

	Df	Sum Sq	Mean Sq	F value	Pr (>F)	
**Species**	4	41031	10257.8	12.3953	1.317e-05	***
**Sites**	7	19955	2850.8	3.4448	0.01076	*
**Residuals**	24	19861	827.6			

CAR

Desiccation

	Df	Sum Sq	Mean Sq	F value	Pr (>F)	
**Species**	4	528.55	132.136	3.6930	0.01763	*
**Sites**	7	519.13	74.161	2.0727	0.08670	
**Residuals**	24	858.73	35.780			

Hydration

	Df	Sum Sq	Mean Sq	F value	Pr (>F)	
**Species**	4	417.06	104.264	4.4552	0.007801	**
**Sites**	7	33.43	4.775	0.2040	0.981354	
**Residuals**	24	521.67	23.403			

Phenolic Compounds

Desiccation

	Df	Sum Sq	Mean Sq	F value	Pr (>F)	
**Species**	4	516835	129209	1.3363	0.285298	
**Sites**	7	2469843	352835	3.6491	0.008041	**
**Residuals**	24	2320569	96690			

Hydration

	Df	Sum Sq	Mean Sq	F value	Pr (>F)	
**Species**	4	9974232	2493558	9.8863	7.134e-05	***
**Sites**	7	8962157	1280308	5.0761	0.001216	**
**Residuals**	24	6053392	252225			

B) Tukey test:

LPX

Desiccation

Species	diff	lwr	upr	p adj
***Po*FIH-*Po*CHF**	-29.70667	-103-37273	43.95939	0.7579847
***Py.orbicularis-Po*CHF**	-44.96667	-100.65297	10.71964	0.1556072
***Py*CHI-*Po*CHF**	120.49667	46.83061	194.16273	0.0005774
***Py*CHJ-*Po*CHF**	-67.30167	-122.98797	-11.61536	0.0124868
***Py.orbicularis-Po*FIH**	-15.26000	-98.78946	68.26946	0.9823585
***Py*CHI-*Po*FIH**	150.20333	53.75182	246.65485	0.0010253
***Py*CHJ-*Po*FIH**	-37.59500	-121.12446	45.93446	0.6782843
Py*CHI-*Py.orbicularis	165.46333	81.93387	248.99280	0.0000466

Py*CHJ-*Py.orbicularis	-22.33500	-90.53652	45.86652	0.8681043
***Py*CHJ-*Py*CHI**	-187.78933	-271.32780	-104.26887	0.0000070

Hydration

Species	diff	lwr	upr	p adj
***Po*FIH-*Po*CHF**	-961.1539	-1883.8178	-38.48998	0.0382883
***Py.orbicularis*-*Po*CHF**	-457.4206	-1154.8889	240.04780	0.3283009
***Py*CHI-*Po*CHF**	1127.9528	205.2889	2050.61669	0.0113382
***Py*CHJ-*Po*CHF**	-752.8039	-1450.2722	-55.33553	0.0299457
***Py.orbicularis*-*Po*FIH**	503.7333	-542.4692	1549.93587	0.6222500
***Py*CHI-*Po*FIH**	2089.1067	881.0560	3297.15730	0.0002908
***Py*CHJ-*Po*FIH**	208.3500	-837.8525	1254.55253	0.9757893
Py*CHI-*Py.orbicularis	1585.3733	539.1708	2631.57587	0.0013928
Py*CHJ-*Py.orbicularis	-295.3833	-1149.6041	558.83746	0.8442556
***Py*CHJ-*Py*CHI**	-1880.7567	-2926.9592	-834.55413	0.0001764

CAR

Desiccation

Species	diff	lwr	upr	p adj
***Po*FIH-*Po*CHF**	1.1488889	-9.840453	12.138231	0.9979019
***Py.orbicularis*-*Po*CHF**	-0.77111111	-9.078273	7.536051	0.9986819
***Py*CHI-*Po*CHF**	13.74222222	2.752880	24.731565	0.0093253
***Py*CHJ-*Po*CHF**	0.07555556	-8.231606	8.382718	0.9999999
***Py.orbicularis*-*Po*FIH**	-1.92000000	-14.380743	10.540743	0.9906707
***Py*CHI-*Po*FIH**	12.59333333	-1.795093	26.981760	0.1065121
***Py*CHJ-*Po*FIH**	-1.07333333	-13.534076	11.387410	0.9990171
Py*CHI-*Py.orbicularis	14.51333333	2.052590	26.974076	0.0168811
Py*CHJ-*Py.orbicularis	0.84666667	-9.327487	11.020821	0.9991418
***Py*CHJ-*Py*CHI**	-13.66666667	-26.127410	-1.205924	0.0266813

Hydration

Species	diff	lwr	upr	p adj
***Po*FIH-*Po*CHF**	-4.508333	-13.3959341	4.3792674	0.5757502

***Py.orbicularis-Po*CHF**	-6.603333	-13.3217280	0.1150613	0.0556861
***Py*CHI-*Po*CHF**	6.603333	-2.6792674	15.09593421	0.2703243
***Py*CHJ-*Po*CHF**	-3.076667	-9.7950613	3.6417280	0.6643755
***Py.orbicularis-Po*FIH**	-2.095000	-12.1725920	7.9825920	0.9717096
***Py*CHI-*Po*FIH**	10.716667	-0.9199342	22.3532676	0.0813883
***Py*CHJ-*Po*FIH**	1.431667	-8.6459253	11.5092587	0.9931469
Py*CHI-*Py.orbicularis	12.811667	2.7340747	22.8892587	0.0080580
Py*CHJ-*Py.orbicularis	3.526667	-4.7016527	11.7549861	0.7156128
***Py*CHJ-*Py*CHI**	-9.285000	-19.3625920	0.7925920	0.0811903

Phenolic Compounds

Desiccation

Species	diff	lwr	upr	p adj
***Po*FIH-*Po*CHF**	120.071111	-451.1989	691.3411	0.9705664
***Py.orbicularis-Po*CHF**	96.526111	-335.3134	528.3656	0.9633156
***Py*CHI-*Po*CHF**	433.567778	-137.7022	1004.8378	0.2009027
***Py*CHJ-*Po*CHF**	9.859444	-421.9801	441.6990	0.9999950
***Py.orbicularis-Po*FIH**	-23.545000	-671.3043	624.2143	0.9999679
***Py*CHI-*Po*FIH**	313.496667	-434.4713	1061.4646	0.7316899
***Py*CHJ-*Po*FIH**	-110.211667	-757.9709	537.5476	0.9864574
Py*CHI-*Py.orbicularis	337.041667	-310.7176	984.8009	0.5522411
Py*CHJ-*Py.orbicularis	-86.666667	-615.5599	442.2266	0.9882360
***Py*CHJ-*Py*CHI**	-423.708333	-1071.4676	224.0509	0.3307797

Hydration

Species	diff	lwr	upr	p adj
***Po*FIH-*Po*CHF**	-961.1539	-1883.8178	-38.48998	0.0382883
***Py.orbicularis-Po*CHF**	-457.4206	-1154.8889	240.04780	0.3283009
***Py*CHI-*Po*CHF**	1127.9528	205.2889	2050.61669	0.0113382
***Py*CHJ-*Po*CHF**	-752.8039	-1450.2722	-55.33553	0.0299457
***Py.orbicularis-Po*FIH**	503.7333	-542.4692	1549.93587	0.6222500
***Py*CHI-*Po*FIH**	2089.1067	881.0560	3297.15730	0.0002908
***Py*CHJ-*Po*FIH**	208.3500	-837.8525	1254.55253	0.9757893

Py*CHI-*Py.orbicularis	1585.3733	539.1708	2631.57587	0.0013928
Py*CHJ-*Py.orbicularis	-295.3833	-1149.6041	558.83746	0.8442556
***Py*CHJ-*Py*CHI**	-1880.7567	-2926.9592	-834.55413	0.001764

Table S4. Two-way ANOVA carried out comparing the effects of treatments (hydration, desiccation, and rehydration) and sites on gene expression *in vitro* of catalase (*cat*), peroxiredoxin (*prx*) and thioredoxin (*trx*) levels for *Porphyra* and *Pyropia* species. *P* values lower than *0.05* indicate significant differences. Df: degrees of freedom; Sum q: Sum square; Mean Sq: Mean square; F: *f* value; Pr(>F): *P* values.

cat

	Df	Sum Sq	Mean Sq	F value	Pr (>F)	
**Treatments**	2	7.2048	3.6024	70.4434	9.043e-11	***
**Sites**	3	0.5870	0.1957	3.8259	0.02263	*
**Tre:Sit**	6	1.8837	0.3140	6.1393	0.0005	***
**Residuals**	24	1.2273	0.0511			

prx

	Df	Sum Sq	Mean Sq	F value	Pr (>F)	
**Treatments**	2	5.9755	2.98775	49.0307	3.339e-09	***
**Sites**	3	0.7583	0.25276	4.1480	0.01675	*
**Tre:Sit**	6	1.9439	0.32398	5.3167	0.00130	**
**Residuals**	24	1.4625	0.06094			

trx

	Df	Sum Sq	Mean Sq	F value	Pr (>F)	
**Treatments**	2	2.1233	1.06167	24.2839	1.712e-06	***
**Sites**	3	0.86984	0.28995	6.6321	0.00202	**
**Tre:Sit**	6	1.04181	0.17363	3.9716	0.00671	**
**Residuals**	24	1.04926	0.04372			

Table S5. Primers sequences used for real-time PCR experiments. Source: [25, 29].

Gene	Abbreviation	Primers sequences	
Catalase	*cat*	F	CGCAGGGATGCCGTAATCAG
		R	CCAGCGACACTTTGTTGACG
Peroxiredoxin	*prx*	F	CTGCCATGCTATGTGACTTTG
		R	CGGCTGCATAATTCGTGAA
Thioredoxin	*trx*	F	CGAATAGGGCAGAGTGTATGA
		R	ACTGTCGCTGATGTGGTTTG
Senescence-associated protein	*sen*	F	TCGCAATGATAGGAAGAGC
		R	TTTACCAGAGGTGTCGGA
